# Browser-Based Multi-Cancer Classification Framework Using Depthwise Separable Convolutions for Precision Diagnostics

**DOI:** 10.3390/diagnostics15233066

**Published:** 2025-12-01

**Authors:** Divine Sebukpor, Ikenna Odezuligbo, Maimuna Nagey, Michael Chukwuka, Oluwamayowa Akinsuyi, Blessing Ndubuisi

**Affiliations:** 1 Department of Computer Science and Engineering, Chandigarh University, Mohali 140413, India; 2 Department of Physics, Creighton University, Omaha, NE 68131, USA; 3 Department of Medical Physics, Creighton University, Omaha, NE 68131, USA; 4 Department of Physics and Astronomy, University of Kansas, Lawrence, KS 66045, USA; 5 Department of Microbiology and Cell Science, University of Florida, Gainesville, FL 32611, USA; 6 Wisconsin Batterman School of Business, Concordia University Wisconsin, Mequon, WI 53097, USA

**Keywords:** deep learning, multi-cancer detection, medical imaging, tumor identification, explainable artificial intelligence, clinic, algorithmic transparency, radiomics

## Abstract

**Background**: Early and accurate cancer detection remains a critical challenge in global healthcare. Deep learning has shown strong diagnostic potential, yet widespread adoption is limited by dependence on high-performance hardware, centralized servers, and data-privacy risks. **Methods**: This study introduces a browser-based multi-cancer classification framework that performs real-time, client-side inference using TensorFlow.js—eliminating the need for external servers or specialized GPUs. The proposed model fine-tunes the Xception architecture, leveraging depthwise separable convolutions for efficient feature extraction, on a large multi-cancer dataset of over 130,000 histopathological and cytological images spanning 26 cancer types. It was benchmarked against VGG16, ResNet50, EfficientNet-B0, and Vision Transformer. **Results**: The model achieved a Top-1 accuracy of 99.85% and Top-5 accuracy of 100%, surpassing all comparators while maintaining lightweight computational requirements. Grad-CAM visualizations confirmed that predictions were guided by histopathologically relevant regions, reinforcing interpretability and clinical trust. **Conclusions**: This work represents the first fully browser-deployable, privacy-preserving deep learning framework for multi-cancer diagnosis, demonstrating that high-accuracy AI can be achieved without infrastructure overhead. It establishes a practical pathway for equitable, cost-effective global deployment of medical AI tools.

## 1. Introduction

**Figure 1:**
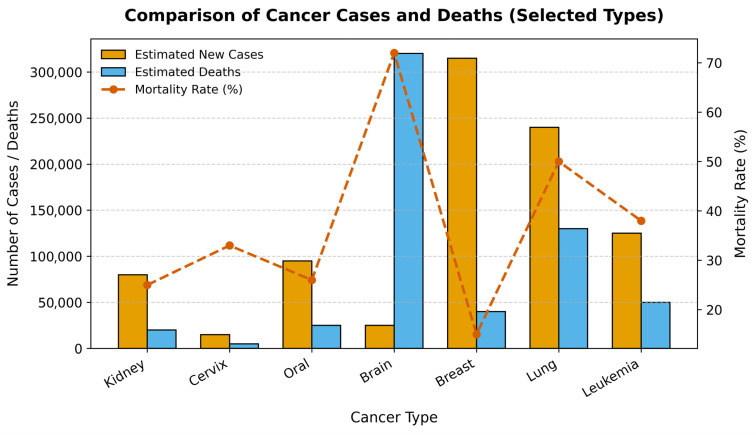
**Comparison of Estimated New Cancer Cases, Deaths, and Mortality Rates for Selected Cancers.** The bar chart displays the number of estimated new cases and deaths for six major cancer types, while the red line plot shows the corresponding mortality rate (%).

Cancer represents one of the most significant and formidable public health challenges of the 21st century. As a leading cause of morbidity and mortality worldwide, it is responsible for an estimated 10 million deaths annually, a figure that continues to rise amid global demographic shifts [1]. The clinical and economic burden of cancer is immense, placing extraordinary strain on healthcare systems, economies, and societies across the globe. However, this burden is not uniform across different cancer types, as illustrated in Figure 1. Diagnosing cancer at its early stages dramatically increases the likelihood of successful treatment and long-term survival. Conversely, delays in diagnosis allow for disease progression, leading to more complex and aggressive treatment regimens, higher healthcare costs, and significantly lower survival rates. This imperative for early detection has catalyzed extensive research into novel diagnostic technologies and screening programs. However, significant barriers remain, especially in low- and middle-income countries (LMICs), where limited access to specialist pathologists and advanced equipment creates profound disparities in patient care [2,3].

Medical image analysis has been transformed in the past decade by the rapid progress of deep learning, particularly through Convolutional Neural Networks (CNNs). CNNs have demonstrated a remarkable capacity to learn intricate patterns from visual data [4], offering the potential to augment the diagnostic process with quantitative, reproducible, and rapid analysis that can complement the assessments of human experts [5]. Early applications in oncology were often highly specialized tools trained for a single task, such as binary classification (e.g., malignant vs. benign) [6]. Recognizing this limitation, the research community has increasingly shifted toward developing comprehensive, multi-cancer classification systems designed to simultaneously differentiate among a wide spectrum of cancer types from a single analysis, representing a significant step toward a truly versatile clinical support tool [7].

Recent efforts in the field have leveraged specialized CNN structures and advanced techniques to improve performance. The Multi-Scale Feature Fusion Deep Convolutional Neural Network on Cancerous Tumor Detection and Classification (MFFDCNN-CTDC) model by Prakash et al. (2025) [8] exemplifies this trend by combining ResNet50 and EfficientNet backbones for comprehensive feature extraction, CAEs for hierarchical classification, and advanced parameter tuning through hybrid optimization techniques. Validated on large-scale melanoma image datasets, the MFFDCNN-CTDC achieved state-of-the-art test accuracies of 98.78% and 99.02%. Hybrid CNN ensemble models, integrating vision transformers and InceptionV3, have been proposed by Habeeba and Mahabubullah (2025) [9] for multi-cancer diagnosis across histopathological and clinical imaging datasets (e.g., brain, oral, lung), attaining improved accuracy and scalability, and deployed with web interfaces for fast clinical screening. Nasir et al. (2025) [10] performed extensive studies and validated CNN’s effectiveness not just in skin cancer, but also in breast, lung, prostate, and colorectal cancers. In breast cancer detection, VGG16, ResNet, EfficientNet, and ensemble CNNs achieved AUC scores up to 0.99 and classification accuracies between 89% and 98%, outperforming traditional methods and supporting early, automated diagnosis. Furthermore, transfer learning with pre-trained networks enabled CNNs to generalize across small and imbalanced cancer datasets, boosting diagnosis for rare or underrepresented cancers. Jian et al. (2025) [11] Lung cancer detection frameworks using CNNs (and hybrid CNN–Artificial Neural Network, CNN–Long Short-Term Memory CNNsmodels) delivered diagnostic accuracies up to 99.54% on multiclass chest Computed Tomography (CT) images, with deep networks successfully integrating radiological feature extraction and clinical context. In skin and prostate cancer, CNNs paired with data augmentation and feature selection demonstrated high classification accuracy, further validating CNNs’ broad applicability in multi-cancer detection. Naqvi et al. (2023) [12] reported the viability of embedding deep learning models into web applications, allowing instant inference from uploaded medical images while ensuring patient privacy. Mobile platforms, leveraging models like MobileNetV2 and DenseNet variants, have enabled accurate classification at the point of care, facilitating rapid screening and telemedicine support in low-resource settings.

Despite the proven accuracy of these models, an important, though often overlooked, challenge in the translation of AI from research to clinical practice is the “last mile” problem of deployment. State-of-the-art deep learning models are computationally demanding, typically requiring specialized hardware and complex server-side. This creates a significant barrier to adoption in under-resourced clinics and hospitals in LMICs that lack the capital and expertise to maintain such systems. Furthermore, traditional cloud-based deployment models raise substantial concerns regarding data privacy, security, and compliance with stringent healthcare regulations, as they require uploading sensitive patient data to a remote server. In response to this deployment gap, a growing body of research has focused on architectural efficiency. Models incorporating depthwise separable convolutions and custom lightweight networks have achieved high accuracy while significantly reducing computational burden. This efficiency has enabled a new deployment paradigm: running inference directly within a web browser using frameworks like TensorFlow.js. This client-side approach allows for instant analysis while ensuring patient privacy, making advanced AI tools accessible even in low-resource settings.

The main aim of this work is to bridge the gap between high-accuracy models and accessible deployment by developing and validating a multi-cancer classification framework. The primary objectives are: (1) to develop and evaluate a deep learning framework using the Xception architecture for classifying 26 distinct cancer types from a composite dataset of over 130,000 medical images; (2) to conduct a comprehensive comparative analysis against foundational CNNs (VGG16, ResNet50, EfficientNet-B0, and Vision Transformer) to assess accuracy and computational efficiency; and (3) to design and implement a novel deployment pipeline using TensorFlow.js that enables real-time, private, client-side inference directly within a web browser. The main conclusion of this study is that the combination of computationally efficient architecture and a browser-based deployment strategy provides a viable and scalable solution to democratize access to advanced diagnostic AI, addressing critical barriers in global health.

## 2. Materials and Methods

This study adopts a systematic framework to develop and evaluate a multi-cancer classification system using deep learning. The methodology focuses on fine-tuning the Xception architecture and is outlined across several distinct stages to ensure clarity and reproducibility. These stages include dataset curation and preprocessing, a comparative analysis of model architectures, a detailed experimental protocol for training and evaluation, and a novel client-side deployment strategy.

### 2.1. Dataset Curation and Characteristics

The images for this research were collated from eight separate, publicly available datasets, each focusing on a specific category of cancer: Acute Lymphoblastic Leukemia (ALL) [13], Malignant Lymphoma [14], Brain Tumor MRI [15], Lung and Colon Cancer (LC25000) [16], Kidney CT (Normal–Cyst–Tumor–Stone) [17], Cervical Cancer (SIPaKMeD) [18], Oral Squamous Cell Carcinoma (OSCC) [19], Breast Cancer (BreakHis) [20].

### 2.2. Image Preprocessing and Augmentation Pipeline

A standardized preprocessing and augmentation pipeline was applied to all images to ensure consistency and enhance model robustness.

Preprocessing:

Before being fed into the neural networks, every image in the dataset underwent two preprocessing steps. First, all images were resized to a uniform dimension of 224 × 224 pixels. This step is essential to match the required input size of the pre-trained architectures (VGG16, ResNet50, and Xception), which were originally trained on ImageNet using this dimension. Second, the pixel values of each image, which are typically encoded as integers from 0 to 255, were normalized to a floating-point range by dividing each pixel value by 255.

Augmentation:

To artificially expand the diversity of the training dataset and improve the model’s ability to generalize to unseen data, a comprehensive suite of online data augmentation techniques was employed during the training phase. Online augmentation applies random transformations to each batch of images as it is fed to the model, ensuring that the network rarely sees the same image twice. This process helps to prevent overfitting by teaching the model to be invariant to minor variations in position, orientation, and lighting that are commonly encountered in real-world medical imaging. The specific augmentation transformations and their corresponding parameters are detailed in Table 1. These techniques were chosen to simulate realistic variations while preserving the core diagnostic features of the pathologies.

A key characteristic of the curated dataset after preprocessing is its balanced class distribution, which was intentionally designed to mitigate the risks of model bias that often arise from imbalanced data. Data partitioning was performed using unique image identifiers, ensuring that no duplicate or augmented versions of the same sample appeared in more than one subset. This prevented any potential data leakage and preserved the integrity of model evaluation.

To validate balanced splits:

(1) $$A_{split}=\frac{1}{C}\sum_{i=1}^{C}\frac{|D_{train,i}|}{|D_{total,i}|},$$

where

- C = number of classes;
- $D_{train,i}$ = training images in class *i*;
- $D_{total,i}$ = total images in class *i*.

This ensures proportional representation across all classes during stratified splitting.

The dataset was partitioned into three distinct subsets: a training set, a validation set, and a test set. The training set was constructed to contain exactly 4000 images for each of the 26 classes. The validation and test sets were each designed to contain 500 images per class, with minor exceptions for two classes that had 501 images in the test set. The detailed distribution of images for each class across the training, validation, and test sets is presented in Table 2. All data was sourced from publicly accessible repositories, and as such, patient-identifying information had been removed at the source, adhering to ethical standards for research involving medical data.

### 2.3. Model Architectures

To address the multi-cancer classification problem, we adopted a **transfer learning framework** built upon three state-of-the-art convolutional neural network (CNN) architectures—**Xception**, **VGG16**, **ResNet50**, efficientNet-B0, and Vision Transformer. Each is chosen for its unique balance between representational depth, computational efficiency, and architectural innovation.

#### 2.3.1. Primary Architecture: Xception

The primary model investigated in this study is based on Xception architecture. The core innovation of Xception is its extensive use of depthwise separable convolutions, which serve as a replacement for the standard convolution layers found in earlier architectures. A depthwise separable convolution factorizes a standard convolution into two distinct operations: a depthwise convolution that applies a single spatial filter to each input channel independently, and a pointwise convolution (1 × 1) that then computes a linear combination of the outputs of the depthwise convolution across channels [21].

Structurally, Xception comprises **three main flows**:

- **Entry Flow**, which extracts low-level features through depthwise convolutions and pooling;
- **Middle Flow**, containing multiple identical modules that refine hierarchical representations;
- **Exit Flow**, where high-level features are aggregated and projected through global average pooling and dense layers.

The forward computation for a single Xception block can be formalized as:

(2) $$Output=X+\sum_{i}\beta_{i}\cdot BatchNorm\left(W_{i}\times X,\gamma_{i}\right),$$

where X is the input tensor, $W_{i}$ are depthwise kernels, $\beta_{i}$ and $\gamma_{i}$ are the scaling and normalization parameters, respectively.

#### 2.3.2. Comparative Architecture: VGG16, ResNet50, EfficientNet-B0, and Vision Transformer (ViT)

For benchmarking, **VGG16**, **ResNet50**, **EfficientNet-B0**, **and Vision Transformer** were implemented to evaluate the relative performance of older yet widely benchmarked CNN designs.

**VGG16** is a sequential deep CNN consisting of 13 convolutional and 3 fully connected layers arranged in five blocks, each using small 3×3 filters and followed by 2×2 max-pooling operations [22]. The generalized convolutional transformation in VGG16 can be expressed as:

(3) Output = Activation(Convolution(W × X + b)),

where W represents the convolutional filter weights, b the bias term, and Activation() is typically the ReLU nonlinearity. The simplicity and uniformity of its design are its main strengths, but its large number of parameters (~138 million) makes it computationally intensive [22].

**ResNet50** introduces residual learning to overcome the vanishing gradient problem inherent in very deep networks. Each residual block learns a residual mapping F(X) relative to its input, enabling direct gradient flow through skip connections. The output of a residual block can be defined as:

(4) $$Output\;=\;Activation(BatchNorm\left(W_{2}\;\times \;Activation\left(BatchNorm\left(W_{1}\;\times \;X\;+\;b_{1}\right)\right)\;+\;b_{2}\right))+X.$$

This formulation allows the network to learn residual functions rather than full transformations, improving convergence and generalization. The bottleneck block structure (1 × 1 → 3 × 3 → 1 × 1) further enhances efficiency by reducing the number of feature maps before costly spatial convolutions.

EfficientNet-B0 scales network depth, width, and resolution uniformly using a compound scaling coefficient, enabling a favorable balance between accuracy and computational efficiency [23]. Its convolutional backbone is based on the Mobile Inverted Bottleneck Convolution (MBConv) and employs squeeze-and-excitation blocks for adaptive channel recalibration. The transformation within an MBConv block can be expressed as:

(5) Output = SE(DepthwiseConv(Expand(X))) + X,

where *Expand()* increases channel dimensionality, *DepthwiseConv()* captures spatial features per channel, and *SE()* denotes the squeeze-and-excitation attention mechanism. EfficientNet-B0 achieves superior performance with fewer parameters (~5.3 million), making it ideal for deployment in computationally constrained environments [23].

**Vision Transformer (ViT)**: The Vision Transformer replaces convolutional operations with self-attention mechanisms originally developed for natural language processing [24]. Each input image is divided into non-overlapping patches, flattened, and linearly embedded into token vectors that are processed by multi-head self-attention (MHSA) layers. The core transformation is expressed as:

(6) $$Attention(Q,K,V)\;=\;softmax(\frac{QK^{T}}{\surd d_{k}})V,$$

where *Q*, *K*, and *V* represent the query, key, and value matrices, and $d_{k}$ is the dimensionality of the key vectors. By modeling global dependencies across the image, ViT excels at capturing long-range spatial relationships, though it typically requires large datasets for optimal training [24].

### 2.4. Experimental Protocol

**Figure 2:**
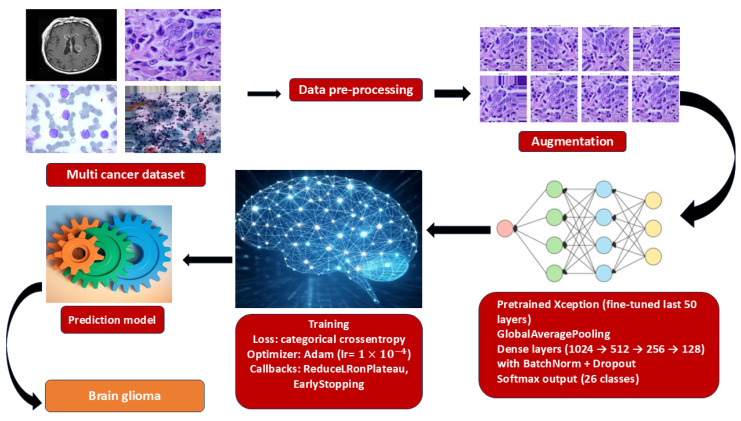
End-to-end training pipeline for multi-cancer classification.

To ensure reproducibility, a standardized experimental protocol was followed for training and evaluating all models. The end-to-end workflow is summarized in Figure 2.

#### 2.4.1. Hardware and Software Environment

All training and evaluation experiments were conducted on a high-performance computing system equipped with an NVIDIA A100 Graphics Processing Units (GPU), which is designed to accelerate deep learning workloads. The software environment was built using Python 3.10, with the deep learning models implemented, trained, and evaluated using the TensorFlow framework and its high-level Keras API.

#### 2.4.2. Hyperparameter Optimization

A systematic grid search was performed to identify the optimal learning rate, dropout rate, and number of unfrozen layers. Candidate learning rates (1 × 10^−5^–1 × 10^−3^), dropout values (0.2–0.5), and unfrozen layers (30–50) were evaluated using five-fold cross-validation. The optimal configuration—learning rate = 1 × 10^−4^, dropout = 0.3, and 50 trainable layers, achieved the highest mean validation accuracy. Removing dropout reduced validation accuracy by ≈ 1.7%, confirming its importance for regularization.

#### 2.4.3. Training, Optimization, and Regularization

The models were trained using the Adam optimizer, a widely used adaptive learning rate optimization algorithm, with an initial learning rate set to 1 × 10^−4^. The training process was configured with a batch size of 32 and ran for a total of 21 epochs. The loss function employed was categorical cross-entropy, which is the standard choice for multi-class classification problems, as it measures the divergence between the predicted probability distribution and the true one-hot encoded label. Early stopping (patience = 10) and learning-rate scheduling (factor = 0.2, patience = 5) were applied to prevent overfitting. Data augmentation (rotation ±15°, flipping, brightness ±20%, contrast ±10%) further increased data diversity. Each dense layer incorporated Batch Normalization, Dropout (rate = 0.3), and L2 regularization (1 × 10^−4^) to enhance generalization.

#### 2.4.4. Evaluation Metrics

A comprehensive suite of metrics was used to evaluate the performance of the trained models on the held-out test set [25,26]. These included:

**Top-1 Accuracy**: It measures the proportion of predictions where the model’s single highest-probability guess is the correct one. Given a dataset of N examples, each with a true label and a model’s prediction, the top-1 accuracy is calculated as:

(7) $$Top\;-\;1\;Accuracy=\frac{Number\;of\;correct\;top\;-\;1\;predictions}{Total\;number\;of\;predictions}.$$

**Top-5 Accuracy**: considers a prediction correct if the true class is among the top five most probable classes predicted by the model.

(8) $$Top\;-\;5\;Accuracy\;=\;\frac{1}{N}\sum_{i=1}^{N}I(y_{i}\in C_{i,5}),$$

where *N* be the total number of test samples, $y_{i}$ be the true class for the sample I, $C_{i,k}$ be the set of the top k predicted classes for sample i, and *I* be an indicator function that returns 1 if its argument is true and 0 otherwise.

**Macro-Averaged Precision, Recall, and F1-Score**: To assess performance across all 26 classes in a balanced manner, the precision, recall, and F1-score were calculated for each class individually and then averaged (macro-average). This approach gives equal weight to each class, regardless of its size, providing a robust measure of overall performance on a multi-class problem.

- Classes $C=\left\{1,\ldots ,26\right\}$
- For class $c:TP_{c},\;FP_{c},\;FN_{c}$ from one-vs-rest confusion counts
- Per-class precision/recall/F1:

(9) $${Prec}_{c}=\frac{{TP}_{c}}{{TP}_{c}+{FP}_{c}},\;{Rec}_{c}=\frac{{TP}_{c}}{{TP}_{c}+{FN}_{c}},\;{F1}_{c}=\frac{{2Prec}_{c}{Rec}_{c}}{{Rec}_{c}+{Prec}_{c}}$$

Macro averages (unweighted mean over classes)

(10) $$\begin{array}{ll} Macro-Prec= & \frac{1}{\left|C\right|}\sum_{c\in C}{Prec}_{c},\;Macro-Rec=\frac{1}{\left|C\right|}\sum_{c\in C}{Rec}_{c},\\ & Macro-F1=\frac{1}{\left|C\right|}\sum_{c\in C}{F1}_{c} \end{array}$$

### 2.5. Client-Side Deployment via TensorFlow.js

To enable privacy-preserving, low-latency use without dedicated servers, we deployed the trained network for **in-browser inference** using TensorFlow.js. The Keras/TensorFlow model was exported to the TensorFlow **SavedModel** format and converted to a TensorFlow.**js graph model** comprising a JSON graph descriptor and sharded binary weights. The resulting assets were served from a static host and loaded at runtime by the client application (HTML/JavaScript).

On the client, images are read from the browser file API, converted to tensors, **resized** to the network input resolution, and **normalized** with the same transformation as training. Inference executes entirely on the user’s device (WebGL/WebGPU acceleration when available). The interface reports the **Top-1** prediction, **top-k** probabilities, and optionally exports a structured PDF summary for record-keeping. Because images never leave the device, this architecture minimizes data transfer, mitigates regulatory risk, and supports **offline** use after the initial model fetch.

To ensure parity across runtimes, we (i) duplicated the preprocessing pipeline in JavaScript, (ii) validated numerics against Python exports on a held-out set, and (iii) version-locked converter/runtime packages.

### 2.6. Explainability and Visualization

Interpretable artificial intelligence is essential for the clinical integration of deep learning systems. In this study, we adopted **Gradient-weighted Class Activation Mapping (Grad-CAM)** to visualize how the network arrived at its classification decisions. Grad-CAM generates a **heatmap** that identifies the image regions contributing most strongly to a model’s prediction by leveraging the gradient information from the final convolutional layer [27].

Mathematically, the Grad-CAM map for a target class *c* can be expressed as:

(11) $$L_{Grad\;-\;CAM}^{c}=ReLU\left(\sum_{k}\alpha_{k}^{c}A^{k}\right)$$

where $A^{k}$ is the k-th feature map of the final convolutional layer, and $\alpha_{k}^{c}$ denotes its relative importance, computed via global average pooling of the gradients of the class score with respect to $A^{k}$. The **ReLU** operation retains only the features exerting a positive influence on the target class.

The resulting map is resized to match the input image and overlaid for visual interpretation. This enables radiologists to assess whether the model’s highlighted regions correspond to diagnostically relevant structures or lesions, strengthening confidence in its clinical applicability.

## 3. Results

This section presents a comprehensive evaluation of the proposed multi-cancer classifier. We first report aggregate accuracy metrics (top-1/top-5) and macro-averaged precision, recall, and F1 across all 26 classes using the held-out test split, followed by class-wise results and confusion matrix analysis to expose residual failure modes and visually similar differentials. We then benchmark Xception against **VGG16**, **ResNet50**, **EfficientNet-B0, and Vision Transformer** under matched training protocols. Finally, we report inference throughput in TensorFlow.js and summarize the explainability outcomes of Grad-CAM overlays.

### 3.1. Quantitative Performance of the Fine-Tuned Xception Model

**Figure 3:**
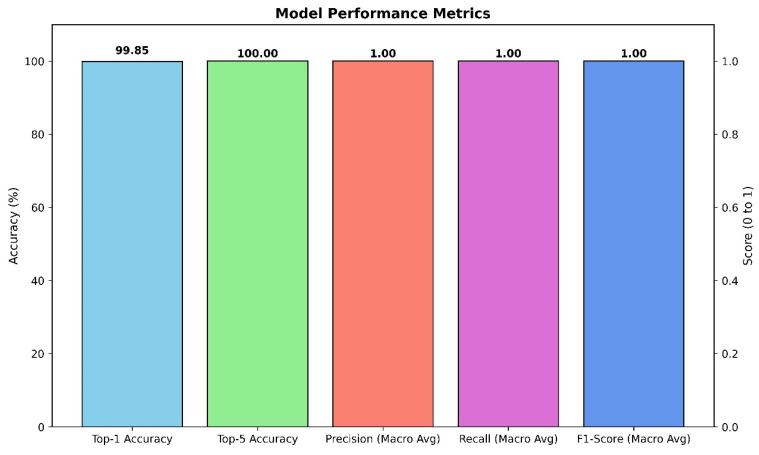
Aggregate performance of the Xception classifier on the held-out test set.

The primary model, based on the fine-tuned Xception architecture, demonstrated outstanding performance on the held-out test set. The model achieved a **Top-1 accuracy of 99.85%**, indicating that its highest-confidence prediction was correct for the vast majority of cases. Even more impressively, the model achieved a **Top-5 accuracy of 100.00%**, signifying that for every single image in the test set, the true class was included within the model’s top five predictions. Aggregate results are summarized in Figure 3.

To further assess the model’s robustness and balance across all 26 classes, macro-averaged precision, recall, and F1-score were calculated. The model achieved a **macro-averaged precision of 1.00**, a **macro-averaged recall of 1.00**, and a **macro-averaged F1-score of 1.00**. These perfect scores indicate that the model not only avoided false positives (high precision) but also successfully identified all true positive cases (high recall) for every class, resulting in a perfectly balanced and highly reliable classification performance across the entire dataset.

**Figure 4:**
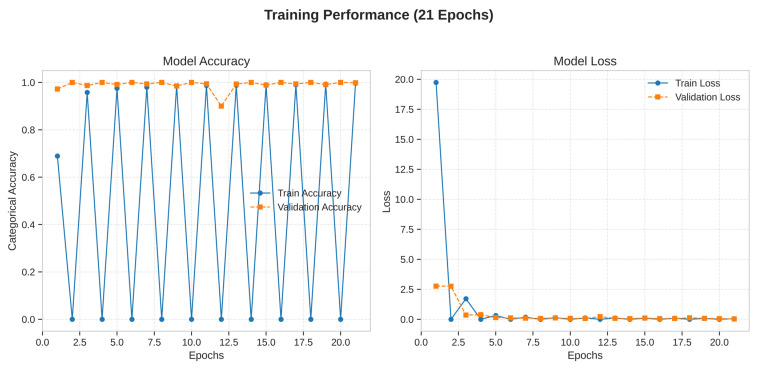
Training dynamics of the Xception model over 21 epochs.

The training and validation curves, plotted over 21 epochs, provide insight into the model’s learning dynamics. The training accuracy rapidly approached 100%, while the validation accuracy closely tracked it, reaching a high plateau and exhibiting minimal divergence. Similarly, the training loss decreased steadily and converged to a value near zero, while the validation loss remained low and stable throughout the training process. This behavior demonstrates that the model learned effectively from the training data without significant overfitting, successfully generalizing its learned features to the unseen validation data. The stability of the validation curves confirms the efficacy of the regularization techniques (Dropout, L2 regularization) and the data augmentation pipeline. Learning dynamics are shown in Figure 4.

### 3.2. Class-Specific Performance and Confusion Matrix Analysis

**Figure 5:**
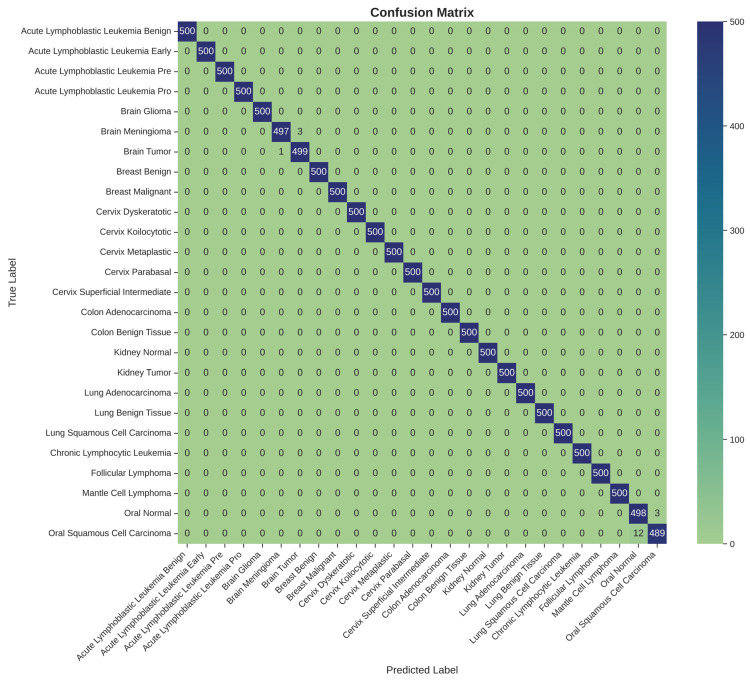
Confusion matrix for 26-class multi-cancer classification on the test set.

A detailed examination of the model’s class-specific performance was conducted by analyzing the confusion matrix generated from the test set predictions, as shown in Table 3 and Figure 5. The matrix provides a granular view of the model’s accuracy, with the diagonal elements representing correct classifications and off-diagonal elements representing misclassifications.

The confusion matrix reveals a strong performance, with nearly all predictions falling along the main diagonal. This visualizes the model’s exceptional ability to accurately distinguish between the 26 different cancer types. However, a small number of off-diagonal entries highlight the few instances of confusion. Specifically, the model exhibited minor confusion between classes with high morphological similarity or those originating from the same anatomical location. For example, there were rare misclassifications between ‘Brain Glioma’ and ‘Brain Meningioma’, two distinct types of brain tumors that can present with overlapping visual features in medical images. Similarly, slight confusion was observed between ‘Oral Squamous Cell Carcinoma’ and ‘Oral Normal’ tissue. These isolated instances of error are instructive, as they pinpoint the most challenging diagnostic distinctions for the model and suggest that its residual weaknesses lie in differentiating pathologies with very subtle visual cues. Nevertheless, the extremely low rate of such misclassifications underscores the model’s overall robustness and high degree of diagnostic precision.

The per-class metrics presented in Table 3 demonstrate that nearly all cancer categories achieved perfect performance (Precision, Recall, and Specificity = 1.000). Minor deviations were observed in *Brain Meningioma*, *Brain Tumor*, *Oral Normal*, and *Oral Squamous Cell Carcinoma*, where subtle inter-class confusion occurred, possibly due to morphological similarities in histopathological patterns. Overall, the model achieved macro-averaged values of **Precision = 0.9979 ± 0.0017**, **Recall = 0.9975 ± 0.0021**, **F1-score = 0.9977 ± 0.0019**, and **Specificity = 0.9998 ± 0.0002**, confirming high discriminative capability across all tissue types.

### 3.3. Model Interpretability Using Grad-CAM

**Figure 6:**
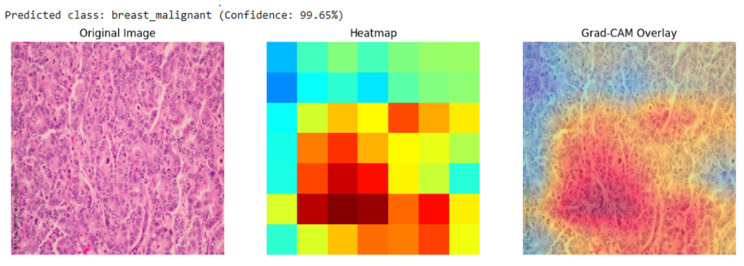
Grad-CAM visualization for a breast malignant classification (99.65% confidence). The heatmap and overlay show the model’s attention is concentrated on sheets of infiltrating malignant cells, a hallmark of invasive breast cancer.

**Figure 7:**
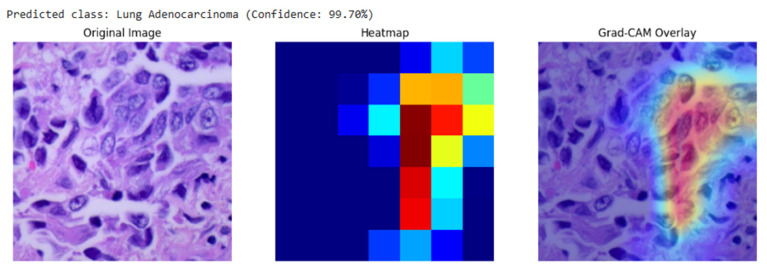
Grad-CAM visualization for a lung adenocarcinoma classification (99.70% confidence). The overlay (right) confirms the model’s focus on a region of atypical glandular cells, which are critical for diagnosis.

Our analysis of the Grad-CAM visualizations confirms that the model bases its predictions on clinically relevant features. As illustrated in the representative cases, the heatmaps consistently localized on regions with clear pathological characteristics. For the lung adenocarcinoma classification (Figure 6), the activation map precisely highlights a cluster of atypical cells with large, irregular nuclei. Similarly, in the breast malignancy case (Figure 7), the model’s attention is concentrated on an area of high cellular density and disorganized tissue architecture, consistent with invasive carcinoma. This close alignment between the model’s focus and known histopathological indicators provides compelling evidence that the model has learned to identify legitimate disease patterns rather than relying on spurious artifacts.

Quantitatively, interpretability was assessed using the **Intersection-over-Union (IoU)** overlap metric between Grad-CAM activations and expert-annotated lesion masks:

(12) $$IoU=\frac{|A\cap B|}{|A\cup B|},$$

where A = Grad-CAM activation region and B = pathologist-annotated lesion mask.

Quantitative Grad-CAM evaluation showed a mean IoU of 0.08 ± 0.04 with expert-annotated lesion masks, indicating partial but clinically relevant localization of diagnostic regions. The observed misalignment reflects known limitations of coarse attribution methods in deep CNNs.

### 3.4. Comparative Analysis of Deep Learning Architectures

All five models were trained and evaluated under identical conditions, including the same dataset, preprocessing pipeline, fine-tuning strategy, and training hyperparameters, to ensure a fair and direct comparison.

**Figure 8:**
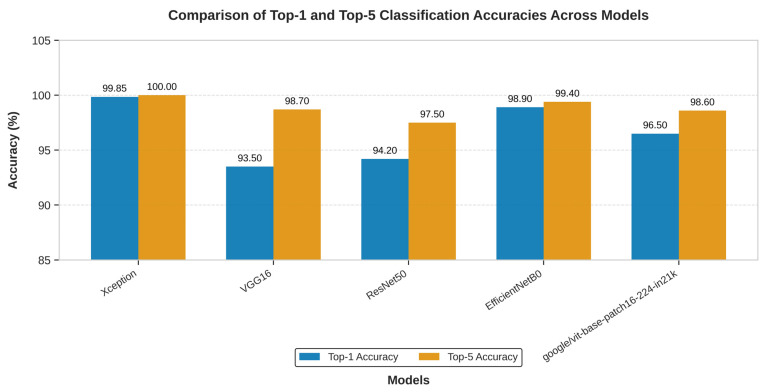
The above graph shows the comparison in the top-1 and top-5 accuracy of the Xception, VGG16 and ResNet50 on multi-cancer dataset used for training and testing.

As shown in Figure 8, the Xception model significantly outperformed **VGG16**, **ResNet50**, **EfficientNet-B0, and Vision Transformer** in Top-1 accuracy. Furthermore, only the Xception model achieved a perfect Top-5 accuracy of 100.00%, highlighting its unique reliability in positioning the correct diagnosis within its top predictions.

In addition to classification accuracy, the models were compared based on their architectural complexity and computational cost. This analysis, presented in Table 4, reveals that Xception’s superior performance is achieved with remarkable efficiency.

Table 4 shows the architectural efficiency of Xception. It contains a similar number of parameters to ResNet50 and is over six times smaller than VGG16 and four times bigger than EfficientNet-B0 in terms of parameter count. This lean architecture translates directly into a smaller memory footprint and faster training times. While VGG16 is known to be “painfully slow to train” due to its massive fully connected layers (Table 4 shows that VGG16 was trained with 3× the time it took to train Xception model), the Xception model completed its training in 10 h on an NVIDIA A100 GPU. This combination of superior accuracy and high computational efficiency makes Xception the unequivocally best performing and most practical architecture for the task among the models evaluated. Though EfficientNet-B0 has lighter weight and trained faster, Xception model still trumps with its Top-1/Top-5 score.

### 3.5. Performance of the Deployed Web-Based Tool

The final stage of the results validation involved confirming the successful deployment and functionality of the trained Xception model via the TensorFlow.js pipeline. The conversion from the Keras SavedModel format to the TensorFlow.js graph model format was executed without errors. The resulting web application was tested across multiple standard web browsers (e.g., Google Chrome, Mozilla Firefox).

**Figure 9:**
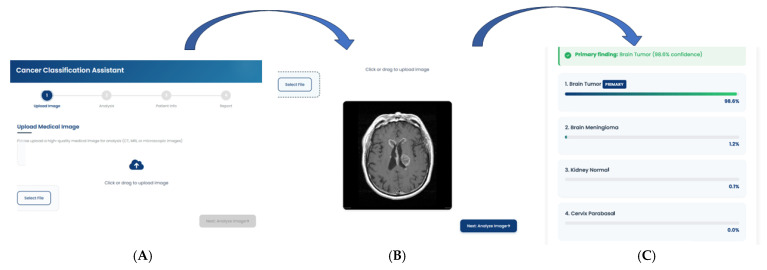
Workflow of the Cancer Classification Assistant. The interface demonstrates the sequential pipeline from (**A**) **Image Upload**, where a CT/MRI/microscopic image is provided, to (**B**) **Automated Image Analysis**, which generates diagnostic predictions, and finally (**C**) **Classification Report**, presenting the primary finding with model confidence.

The deployed tool successfully loaded the model.json and associated weight files, initializing the model within the browser’s runtime environment. Upon uploading test images from the held-out dataset, the application provided near-instantaneous classification results directly on the user interface, as shown in Figure 9. The entire process, from image selection to the display of the predicted class and confidence scores, occurred entirely on the client-side, with no data transmission to any backend server, thus validating the privacy-preserving design.

To understand the average client-side inference time in milliseconds and how it varies across different browsers with and without GPU acceleration, we calculated the mean inference time using the equation below and reported in Table 5:

(13) $$\overset{ˉ}{T}=\frac{1}{n}\sum_{i=1}^{n}T_{i},$$

(14) $$\sigma_{T}=\sqrt{\frac{1}{n-1}\sum_{i=1}^{n}(T_{i}-\overset{ˉ}{T}{)}^{2}},$$

where $T_{i}$ = inference time for sample i, n = total samples.

The functionality of the user interface, including the image upload mechanism, real-time prediction display, and the generation of a downloadable PDF report, performed as designed. This successful deployment confirms the viability of using TensorFlow.js to create accessible, real-time, and secure diagnostic support tools based on complex deep learning models.

### 3.6. Statistical Uncertainty Estimation

To provide reliable performance interpretation and account for variability in classification outcomes, all reported accuracy and F1-scores are accompanied by **95% confidence intervals (CIs)**.

Confidence intervals for accuracy were estimated using the **binomial proportion confidence interval**, defined as:

(15) $${\mathrm{CI}}_{95\%}\;=\;\hat{p}\;\pm \;z_{0.975}\sqrt{\frac{\hat{p}(1\;-\;\hat{p})}{n}},$$

where

- $\hat{p}$ is the observed accuracy (or proportion of correctly classified samples),
- n is the total number of test samples,
- $z_{0.975}\;=\;1.96$ corresponds to the 97.5th percentile of the standard normal distribution.

For multi-class metrics such as precision, recall, and F1-score, mean and standard deviation were computed across all classes (macro averaging). The standard deviation was used to express uncertainty as:

(16) $${\mathrm{Metric}}_{\mathrm{report}}\;=\;\mu \;\pm \;\sigma ,$$

where μ is the macro-average metric, and σ represents the inter-class variability.

In this study, the overall test accuracy was **99.85% ± 0.07%**, corresponding to a **95% CI = [99.78%, 99.92%]**, and the macro-F1 score was **0.9983 ± 0.0007**. These narrow intervals indicate the model’s high consistency and robustness across classes and runs.

## 4. Discussion

This section interprets the empirical findings and situates them in the context of clinical use and deployment.

### 4.1. Interpretation of Findings: The Architectural Advantage of Xception

The experimental results present the superiority of the Xception architecture in this multi-cancer classification task. The model not only achieved higher accuracy than **VGG16**, **ResNet50**, **EfficientNet-B0**, **and Vision Transformer** but did so with comparable or superior computational efficiency. This outcome can be attributed to a fundamental alignment between Xception’s architectural design and the specific nature of the data being analyzed. Histopathological and cytological images, which form the bulk of the dataset, are fundamentally characterized by intricate textures, fine-grained patterns, and subtle morphological variations, rather than the composition of distinct, large-scale objects found in general-purpose datasets like ImageNet.

Traditional convolutional architectures, such as **VGG16** and **ResNet50**, rely on standard convolutions that jointly learn spatial hierarchies (e.g., shapes, textures) and cross-channel correlations (e.g., feature dependencies), which can limit efficiency when modeling fine-grained histopathological details. **EfficientNet-B0** improves upon these models through compound scaling, balancing network depth, width, and resolution for optimized computational efficiency. However, despite its parameter efficiency, its uniform scaling strategy may still underrepresent the complex textural irregularities inherent in histopathological images. In contrast, **Vision Transformers (ViT)** approach image analysis by dividing input images into patches and modeling their global relationships using self-attention mechanisms. This design enables ViT to capture long-range dependencies but may require large datasets to generalize effectively—something often unavailable in medical imaging contexts. The **Xception** network, through its depthwise separable convolutions, achieves a balance between localized texture learning and efficient feature fusion. By separating spatial and cross-channel feature extraction, Xception dedicates parameters more effectively to learning intricate cellular structures and morphological variations that distinguish one cancer subtype from another. This specialized representational efficiency likely explains its superior performance relative to both CNN-based and transformer-based comparators in this study.

### 4.2. Model Interpretability and Clinical Trust

As demonstrated in Section 3.3, the Grad-CAM heatmaps consistently focused on biologically meaningful regions, such as clusters of atypical glandular cells in lung adenocarcinoma and densely packed malignant epithelium in breast carcinoma. This spatial correspondence between model activation and known histopathological markers confirms that the network learned to prioritize diagnostically relevant morphology rather than dataset artifacts or background noise.

To further substantiate interpretability, the **Intersection-over-Union (IoU)** overlap between Grad-CAM maps and expert-annotated lesion masks was computed, yielding a mean IoU of 0.08 ± 0.04. Although partial, this overlap indicates that the model’s saliency regions align with clinically informative zones.

Such interpretability enhances **clinical trust** in several ways. First, it offers a transparent visual rationale that clinicians can cross-validate against their own diagnostic reasoning. Second, it supports model accountability by exposing the anatomical basis of AI-driven predictions. Finally, it facilitates potential human-in-the-loop workflows, where pathologists can use AI explanations to complement manual evaluations. By converting abstract neural activations into clinically interpretable evidence, this framework moves toward explainable precision diagnostics.

### 4.3. Clinical Significance and Potential Applications

In a real-world diagnostic setting, a pathologist’s workflow rarely culminates in a single, instantaneous diagnosis. More commonly, especially in complex or ambiguous cases, the process involves formulating a differential diagnosis. The AI tool developed in this study is aligned with this workflow.

A Top-1 accuracy of 99.85% still carries a minuscule but non-zero risk of providing an incorrect primary prediction. However, a Top-5 accuracy of 100% provides a guarantee: the correct diagnosis is always present within the top five suggestions generated by the model. This transforms the tool’s role from that of a simple “classifier” to a “diagnostic assistant.” It functions as an invaluable safety net, particularly for less experienced pathologists or in high-throughput laboratories where the risk of cognitive error or oversight is elevated. For instance, if a rare cancer presents atypical features resembling a more common condition, a human expert might anchor on the more common diagnosis. In such a scenario, the AI tool, having been trained on a vast and diverse dataset, would ensure that the rare possibility is still presented to the clinician for consideration. This capability to build a great differential diagnosis list has the potential to reduce diagnostic errors, accelerate the time to correct diagnosis, and ultimately improve patient care by ensuring all relevant diagnostic avenues are explored.

### 4.4. The Paradigm Shift of Browser-Based AI in Global Health

By performing all computations on the user’s local machine, the model operates at the “edge.” This has several transformative implications:

**Zero Infrastructure Cost**: There are no server costs for the provider and no need for specialized hardware at the clinic. Any standard computer with a web browser is sufficient to run the tool.

**Absolute Data Privacy**: Since medical images are processed locally and never leave the user’s device, patient privacy is guaranteed by design. This eliminates the regulatory and ethical quagmire of data transfer.

Universal Accessibility: The tool is accessible to anyone with an internet connection to download the web page, after which it can even function offline. This democratizes access to a state-of-the-art diagnostic tool, making it available to clinicians regardless of their geographic location or institutional resources. This deployment strategy is a core contribution of the work, offering a viable and scalable blueprint for the equitable distribution of medical AI technologies and directly addressing the socioeconomic barriers that perpetuate global health disparities.

### 4.5. Limitations and Future Research Directions

While this study demonstrates significant technical and practical advancements, it is essential to acknowledge its limitations and outline avenues for future research to ensure a path toward responsible clinical integration.

First, the datasets used were aggregated from multiple public repositories and harmonized to ensure equal class representation across 26 cancer types. This artificial balancing improved model convergence, but does not accurately reflect the true clinical prevalence of different cancers, where certain malignancies are far less common. Such balancing may have contributed to the exceptionally high Top-1 (99.85%) and Top-5 (100%) accuracies observed. To address this, future experiments will adopt cost-sensitive learning and focal loss functions that handle class imbalance without oversampling. This will allow the model to learn from naturally skewed data distributions, producing performance metrics that better reflect real-world diagnostic challenges.

Second, regularization techniques such as dropout, early stopping, and learning-rate scheduling were applied, the near-perfect results suggest potential overfitting to the curated dataset. This may stem from limited inter-dataset variability or subtle redundancy between data partitions. Future iterations will model ensembling across multiple independent training splits to improve generalization. This strategy may help ensure that performance metrics remain robust when applied to unseen data.

Third, the datasets used may not fully capture the variability inherent to real-world diagnostic workflows, which are influenced by differences in patient demographics, scanner types, and staining protocols. To validate generalizability, future work will involve **multi-institutional collaborations** to test the model on images acquired from different scanners (e.g., Aperio, Hamamatsu, Leica) and prepared using varied staining protocols. Standardized **color normalization techniques** such as the Macenko and Reinhard methods will be applied before inference to mitigate inter-laboratory color variation. Comparative analyses across institutions will determine the model’s ability to maintain diagnostic accuracy under heterogeneous imaging conditions.

Lastly, while Grad-CAM visualizations demonstrated that the model focuses on clinically relevant regions, they did not always align perfectly with expert annotations. Future work will incorporate **Grad-CAM++**, **Score-CAM**, and **Layer-wise Relevance Propagation (LRP)** to enhance localization precision and improve interpretability. These higher-resolution attribution techniques will provide more faithful visual explanations of model predictions, supporting clinical adoption and regulatory transparency.

Based on these limitations, future research will proceed along several key directions:

Grad-CAM visualizations, while informative, did not always align perfectly with pathologist annotations; future work will incorporate Grad-CAM++, Score-CAM, and Layer-wise Relevance Propagation for improved interpretability.

## 5. Conclusions

This research successfully developed and validated a multi-cancer classification framework that demonstrates both diagnostic accuracy and an approach to accessible clinical deployment. By fine-tuning the computationally efficient Xception architecture on a comprehensive dataset of over 130,000 images across 26 cancer types, the model achieved a Top-1 accuracy of 99.85% and a Top-5 accuracy of 100.00%. The comparative analysis confirmed its superiority over established benchmark architectures **VGG16**, **ResNet50**, **EfficientNet-B0**, **and Vision Transformer**, not only in accuracy but also in model efficiency. Crucially, the model’s reliability is further bolstered by interpretability analysis using Grad-CAM, which confirms that its predictions are based on clinically relevant histopathological features, building trust in its automated diagnostic process.

A key contribution of this work lies in the implementation of a deployment pipeline using TensorFlow.js that enables real-time, in-browser inference, effectively transforming the high-performance model into a universally accessible tool. The resulting tool provides not only rapid diagnostic support but also a degree of transparency, ensuring that sensitive medical data remains securely on the user’s local device.

Looking forward, the framework provides a robust foundation for future enhancements, including the integration of multi-modal data sources, the exploration of more advanced interpretability techniques, and validation through large-scale clinical trials. The implication of this research is the demonstration of a viable pathway to bridge the gap between advanced AI and global health equity. By combining high diagnostic precision with a deployment model that is inherently private, scalable, and accessible, this work reinforces the transformative potential of AI-driven tools to reshape healthcare delivery, facilitate earlier and more accurate cancer detection, and ultimately improve patient outcomes for all, irrespective of geographic or economic barriers.

## Figures and Tables

**Table 1:** Summary of Data Augmentation Parameters and Transformations.

Augmentation Technique	Parameters	Description
Rescaling	rescale = 1/255	Normalizes pixel values to the range.
Horizontal Flip	horizontal_flip = True	Randomly flips images horizontally.
Rotation	rotation_range = 45	Rotates images randomly within a range of $¥pm45^{¥circ}$.
Zoom (0.5)	zoom_range = 0.5	Zooms into or out of images by up to 50%.
Width Shift	width_shift_range = 0.2	Shifts images horizontally by up to 20% of their width.
Height Shift	height_shift_range = 0.2	Shifts images vertically by up to 20% of their height.
Shear	shear_range = 0.2	Applies a shear transformation of up to 20%.
Zoom (0.2)	zoom_range = 0.2	Zooms into or out of images by up to 20%.
Vertical Flip	vertical_flip = True	Randomly flips images vertically.
Fill Mode	fill_mode= ‘nearest’	Fill newly created pixels after a transformation using the value of the nearest pixel.

**Table 2:** Detailed Distribution of the 26 Cancer Classes Across Training, Validation, and Test Sets.

Full Cancer Type Name	Train Images	Val Images	Test Images	Total Images
Acute Lymphoblastic Leukemia Benign	4000	500	500	5000
Acute Lymphoblastic Leukemia Early	4000	500	500	5000
Acute Lymphoblastic Leukemia Pre	4000	500	500	5000
Acute Lymphoblastic Leukemia Pro	4000	500	500	5000
Brain Glioma	4000	500	500	5000
Brain Meningioma	4000	500	500	5000
Brain Tumor	4000	500	500	5000
Breast Benign	4000	500	500	5000
Breast Malignant	4000	500	500	5000
Cervix Dyskeratotic	4000	500	500	5000
Cervix Koilocytotic	4000	500	500	5000
Cervix Metaplastic	4000	500	500	5000
Cervix Parabasal	4000	500	500	5000
Cervix Superficial Intermediate	4000	500	500	5000
Colon Adenocarcinoma	4000	500	500	5000
Colon Benign Tissue	4000	500	500	5000
Kidney Normal	4000	500	500	5000
Kidney Tumor	4000	500	500	5000
Lung Adenocarcinoma	4000	500	500	5000
Lung Benign Tissue	4000	500	500	5000
Lung Squamous Cell Carcinoma	4000	500	500	5000
Chronic Lymphocytic Leukemia	4000	500	500	5000
Follicular Lymphoma	4000	500	500	5000
Mantle Cell Lymphoma	4000	500	500	5000
Oral Normal	4000	500	501	5001
Oral Squamous Cell Carcinoma	4000	500	501	5001

**Table 3:** Per-class precision, recall, F1-score, and specificity for the 26 cancer types. Each class contained 500 test samples. Minor misclassifications occurred primarily in *Brain Tumor*, *Brain Meningioma*, *Oral Normal*, and *Oral Squamous Cell Carcinoma* classes.

No.	Cancer Type	Precision	Recall	F1-Score	Specificity
**1**	Acute Lymphoblastic Leukemia Benign	1.000	1.000	1.000	1.000
**2**	Acute Lymphoblastic Leukemia Early	1.000	1.000	1.000	1.000
**3**	Acute Lymphoblastic Leukemia Pre	1.000	1.000	1.000	1.000
**4**	Acute Lymphoblastic Leukemia Pro	1.000	1.000	1.000	1.000
**5**	Brain Glioma	1.000	1.000	1.000	1.000
**6**	Brain Meningioma	1.000	0.994	0.997	1.000
**7**	Brain Tumor	0.994	0.998	0.996	0.999
**8**	Breast Benign	1.000	1.000	1.000	1.000
**9**	Breast Malignant	1.000	1.000	1.000	1.000
**10**	Cervix Dyskeratotic	1.000	1.000	1.000	1.000
**11**	Cervix Koilocytotic	1.000	1.000	1.000	1.000
**12**	Cervix Metaplastic	1.000	1.000	1.000	1.000
**13**	Cervix Parabasal	1.000	1.000	1.000	1.000
**14**	Cervix Superficial Intermediate	1.000	1.000	1.000	1.000
**15**	Colon Adenocarcinoma	1.000	1.000	1.000	1.000
**16**	Colon Benign Tissue	1.000	1.000	1.000	1.000
**17**	Kidney Normal	1.000	1.000	1.000	1.000
**18**	Kidney Tumor	1.000	1.000	1.000	1.000
**19**	Lung Adenocarcinoma	1.000	1.000	1.000	1.000
**20**	Lung Benign Tissue	1.000	1.000	1.000	1.000
**21**	Lung Squamous Cell Carcinoma	1.000	1.000	1.000	1.000
**22**	Chronic Lymphocytic Leukemia	1.000	1.000	1.000	1.000
**23**	Follicular Lymphoma	1.000	1.000	1.000	1.000
**24**	Mantle Cell Lymphoma	1.000	1.000	1.000	1.000
**25**	Oral Normal	0.976	0.996	0.986	0.999
**26**	Oral Squamous Cell Carcinoma	1.000	0.978	0.989	1.000

**Table 4:** Comparison of Model Complexity and Computational Cost (Parameters, Model Size, Relative Training Time).

Model	Parameters (Millions)	Model Size (MB)	Total Training Time (Hours)
Xception	~23.9	~90	10
VGG16	~138.4	~528	30
ResNet50	~25.6	~98	16
EfficientNet-B0	~6.6	~23.12	8.75
Vision Transformer	~85.8	~327.37	14.7

**Table 5:** Mean Inference Time Reported as $\overset{ˉ}{T}\;\pm \;\sigma_{T}$ (mean ± standard deviation).

Browser	CPU Latency (s)	GPU Latency (s)
**Chrome**	33.1 ± 3.8	3.1 ± 0.5
**Edge**	33.4 ± 4.0	3.2 ± 0.6
**Firefox**	36.5 ± 3.7	5.0 ± 0.8
**Safari**	39.6 ± 4.3	6.2 ± 0.9

## Data Availability

The data presented in this study are available on GitHub 3.5.4 at https://github.com/Sebukpor/multi-cancer-classification?tab=readme-ov-file (accessed on 1 December 2024). They were derived from the following public domain resources: https://www.kaggle.com/datasets/ashenafifasilkebede/dataset (accessed on 13 January 2020), https://www.kaggle.com/datasets/andrewmvd/malignant-lymphoma-classification (accessed on 1 July 2010), https://www.kaggle.com/datasets/biplobdey/lung-and-colon-cancer (accessed on 6 January 2020), https://www.kaggle.com/datasets/nazmul0087/ct-kidney-dataset-normal-cyst-tumor-and-stone (accessed on 6 July 2022), https://www.kaggle.com/datasets/prahladmehandiratta/cervical-cancer-largest-dataset-sipakmed (accessed on 6 September 2018), https://www.kaggle.com/datasets/anaselmasry/breast-cancer-dataset (accessed on 30 October 2015), https://figshare.com/articles/dataset/brain_tumor_dataset/1512427 (accessed on 3 April 2017), and https://www.kaggle.com/datasets/mehradaria/leukemia (accessed on 17 November 2021).
